# LncRNA as ceRNAs may be involved in lactation process

**DOI:** 10.18632/oncotarget.20439

**Published:** 2017-08-24

**Authors:** Shuai Yu, Yong Zhao, Fangnong Lai, Meiqiang Chu, Yanan Hao, Yanni Feng, Hongfu Zhang, Jing Liu, Ming Cheng, Lan Li, Wei Shen, Lingjiang Min

**Affiliations:** ^1^ College of Animal Science and Technology, Qingdao Agricultural University, Qingdao, P. R. China; ^2^ State Key Laboratory of Animal Nutrition, Institute of Animal Sciences, Chinese Academy of Agricultural Sciences, Beijing, P.R. China; ^3^ Core Laboratories of Qingdao Agricultural University, Qingdao, P. R. China; ^4^ Qingdao Veterinary and Livestock Administration, Qingdao, P.R. China

**Keywords:** milk, lactation, ceRNA, lncRNA-mRNA, correlation

## Abstract

The main function of the mammary gland is to secret milk for newborn growth. Milk production process is regulated by hormones, growth factors, noncoding RNAs and other factors locally. Long non-coding RNAs (lncRNAs), one type of recently discovered non-coding RNA, have been found in mammary gland and some studies suggested lncRNA may play important roles in mammary gland development. Competing endogenous RNAs (ceRNAs) are emerging to compete for miRNA binding and, in turn, regulate each other. In the current study, we sequenced mRNA, miRNA and lncRNA in goat mammary tissue at 2 points in lactation (early and mature). All data were co-expressed together from the same samples. Our data showed that the ceRNAs up-regulated during the mature lactation phase were associated with lipid, protein, carbon and amino acid synthesis and metabolism. This correlates with the function of the mature lactation phase: i.e. the continuous production of large amounts of milk, rich in proteins, lipids, amino acids and other nutrients. Alternately, the ceRNAs up-regulated during early lactation were associated with PI3K-AKT pathways and ECM-receptor interactions; these fulfil the functional role of preparing the mammary gland for full lactation. Therefore, the results suggest that ceRNAs work synergistically during different developmental stages to regulate specific functions associated with lactation control. This study suggests that ceRNAs (lncRNA-mRNA) may be involved in lactation process.

## INTRODUCTION

Milk contains crucial biologically active components for infant growth and development, including immunological, gastrointestinal, neural and even intellectual development [1]. Mammary gland is a complex gland that originates during the embryonic stage and develops quickly from the pubertal stage with ductal growth and early alveolar development during menstrual cycles; mammary tissue then undergoes proliferation, differentiation and death during pregnancy, lactation and involution stages, respectively [2]. The main function of the mammary gland is to secret milk for infant nutrition [3–10].

The regulation of milk synthesis impacts on the health of both the mother and the neonate [11–13]. Anderson *et al*. [13] reports four phases for the mammary gland according to its function: proliferation phase during early pregnancy; secretory differentiation phase starting from mid-pregnancy; secretory activation phase beginning around parturition; and lactation phase associated with continued milk production. Lactation itself includes 2 phases: early phase (un-mature phase) when milk is composed of large amounts of immuno-factors however with less volume; and the mature secretion phase when large volumes of milk are continuously produced to support growth and development of the newborn [13]. Milk production is an evolutionary necessity and this process is tightly regulated at a local level, i.e. within the mammary gland itself, by hormones and other factors [7–10].

Elucidation of the molecular events of mammary gland development during lactation can aid understanding of molecular development from theearly phase (un-mature phase) to the mature secretion phase and could help to improve milk production [14]. Therefore, identifying the regulatory principles that govern mammary gland development during lactation has attracted the attention of developmental, molecular biologists and even clinicians [14]. MicroRNAs (miRNAs), a class of small noncoding RNAs, have been broadly investigated in mammary gland development due to their inhibitory effects on their target genes [15–18]. Recently, competing endogenous RNAs (ceRNAs), sharing miRNA recognition elements (MREs), are emerging to compete for miRNA binding and, in turn, regulate each other. Interactions of ceRNAs have potential roles in gene expression and cell phenotypes [19, 20]. Nowadays, long non-coding RNAs (lncRNAs), one type of recently discovered non-coding RNA, act as ceRNAs to regulate gene expression [21–23]. Furthermore, the important roles of lncRNAs in mammary gland development are becoming increasingly evident [24–28]. Standaert *et al*. [28] identified that the lncRNA Neat1 plays an important role in mammary gland cell proliferation during lobular-alveolar development. However, these studies simply investigated the expression of lncRNA in the mammary gland but no ceRNAs of lncRNA-mRNA (based on miRNA) were explored. Moreover, the roles of lncRNA associated-ceRNAs in lactation, particularly in the transition from the early (un-mature) phase to the mature secretion phase, are not fully understood. Moreover, the dynamic patterns of ceRNA interactions during this developmental process remain unknown. To better understand the dynamic process of mammary gland development during early lactation, RNA-sequencing of mammary tissue at different time periods during lactation was performed to analyse mRNA, lncRNA and miRNA expression. Furthermore, ceRNA networks (ceR NETs) were established for protein coding genes and lncRNAs.

## RESULTS

### Morphologic changes in mammary gland tissue and changes in expression of mRNAs, miRNAs and lncRNAs during lactation

**Figure 1:**
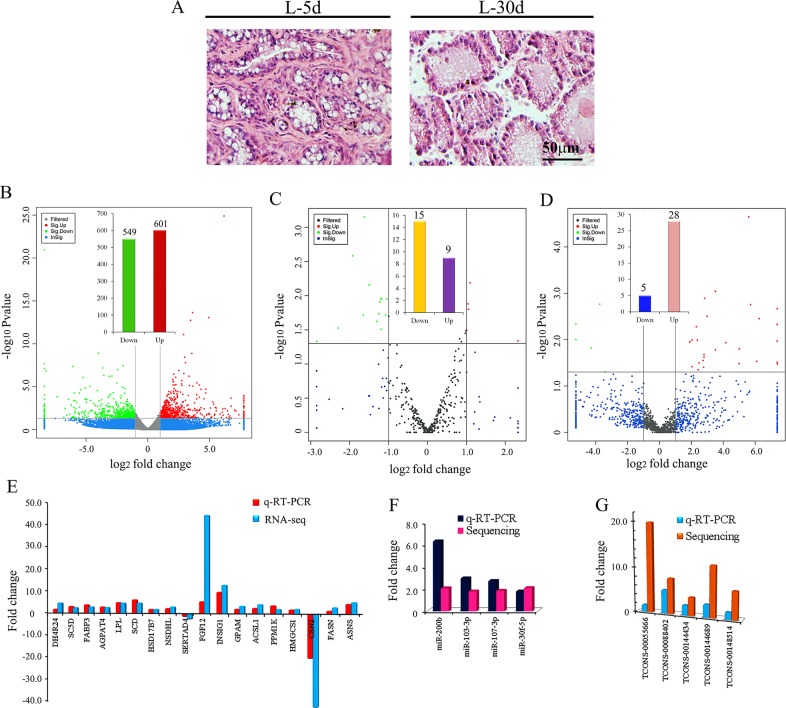
**(A)** Histological features of the mammary gland of goat during early (L-5 d) and mature (L-30 d) lactation phases. **(B)** The expression of mRNA by RNA-seq using Volcano photo. The green dots show the down-regulated mRNA at L-30 d; and the red dots shows the up-regulated mRNA at L-30 d. **(C)** The expression of miRNA by small RNA sequencing using Volcano photo. The green dots show the down-regulated miRNA at L-30 d; and the red dots show the up-regulated miRNA at L-30 d. **(D)** The expression of lncRNA by RNA-seq using Volcano photo. The green dots show the down-regulated lncRNA at L-30 d; and the red dots show the up-regulated lncRNA at L-30 d. **(E)** The comparing data of q-RT-PCR and sequencing for 18 mRNAs. **(F)**. The comparing data of q-RT-PCR and sequencing for 4 miRNAs. **(G)** The comparing data of q-RT-PCR and sequencing for 5 lncRNAs.

Histological changes in goat mammary gland morphology during early lactation (L-5 d) and peak lactation (L-30 d) are shown in Figure 1A. At L-5 d, which represented the colostral phase, the luminal space of the lobuloalveolar structures expanded and the epithelial cell layer was prominent against the adipocytes (Figure 1A left). At L-30 d, which represented the mature secretion phase, the goat mammary gland was stably producing copious amounts of milk and exhibited prominent luminal structures and ducts with only a few adipocytes (Figure 1A right). Adipocytes are known to become de-lipided with no change in cell number [13, 29]. Goat milk contains about 3.5% protein (caseins, whey acidic protein (WAP), lactoferrin, secretory immunoglobulin A, and others), 3.8% lipid and 4.1% lactose, a disaccharide that is unique to milk [30, 31].

To systematically detect the expression of coding genes (mRNA), microRNAs and lncRNAs during goat lactation, we first analysed the expression profiles of RNAs by RNA-seq and small RNA sequencing for goat mammary gland tissues from L-5 d and L-30 d (Supplementary Table 1, Supplementary Table 2, Supplementary Table 3 and Supplementary Table 4). In order to search for the genes, miRNAs or lncRNAs related to lactation, the data at L-5 d was considered as basal (control) and compared with data at L-30 d. In total, 42 690 mRNAs were found in mammary gland tissues of which 1150 genes were differentially expressed at L-30 d (549 down- and 601 up-regulated; Figure 1B). The expression of 18 genes was confirmed by real time quantitative RT-PCR (Figure 1E). The q-RT-PCR data and the RNA-seq data were in the similar trend. In total, 391 known miRNAs were detected in the mammary tissues of which 24 known miRNAs were differentially expressed (15 down- and 9 up-regulated; Figure 1C; Table 1). The expression of 4 miRNAs was confirmed by real time quantitative RT-PCR (Figure 1F). The q-RT-PCR data and the RNA-seq data were similar. In addition, 985 novel miRNAs were found in the mammary tissue of which 7 novel miRNAs were down- and 10 differentially up-regulated (Supplementary Table 5, Supplementary Table 6). In total, 1451 lncRNAs were detected in the mammary tissue of which 33 were differentially expressed at L-30 d (5 down- and 28 up-regulated; Figure 1D; Table 2). The expression of 5 lncRNAs was confirmed by real time quantitative RT-PCR (Figure 1F). The q-RT-PCR data for lncRNAs was similar to the RNA-seq data.

**Table 1:** The differentially expressed known miRNAs

miRNA	foldChange	log2FoldChange	pval	padj	up_down	Sequence
**chi-miR-491-3p**	5.01	2.33	0.046	0.769	Up	CTTATGCAAGATTCCCTTCTAC
**chi-miR-200c**	2.14	1.10	0.006	0.559	Up	TAATACTGCCGGGTAATGATGGA
**chi-miR-30f-5p**	2.08	1.05	0.016	0.559	Up	TGTAAACACCCTACACTCTCAGC
**chi-miR-200b**	2.06	1.04	0.013	0.559	Up	TAATACTGCCTGGTAATGATGA
**chi-miR-378-5p**	2.01	1.01	0.032	0.671	Up	CTCCTGACTCCAGGTCCTGTGT
**chi-miR-22-3p**	1.98	0.99	0.033	0.671	Up	AAGCTGCCAGTTGAAGAAC
**chi-miR-3431-5p**	1.96	0.97	0.035	0.691	Up	CCTCAGTCAGCCTTGTGGATGT
**chi-miR-107-3p**	1.83	0.87	0.047	0.769	Up	AGCAGCATTGTACAGGGCTAT
**chi-miR-103-3p**	1.78	0.83	0.043	0.769	Up	AGCAGCATTGTACAGGGCTATGA
**chi-miR-204-5p**	0.50	-1.00	0.020	0.559	Down	TTCCCTTTGTCATCCTATGCCT
**chi-miR-301a-3p**	0.48	-1.05	0.011	0.559	Down	CAGTGCAATAGTATTGTCAAAGC
**chi-miR-432-5p**	0.48	-1.07	0.019	0.559	Down	TCTTGGAGTAGGTCATTGGGTGG
**chi-miR-454-3p**	0.44	-1.19	0.011	0.559	Down	TAGTGCAATATTGCTTATAGGGT
**chi-miR-502b-3p**	0.44	-1.19	0.031	0.671	Down	ATCCACCTGGGCAAGGATTCTGAA
**chi-miR-376a**	0.44	-1.20	0.011	0.559	Down	GTAGATTCTCCTTCTATGAGT
**chi-miR-542-5p**	0.43	-1.22	0.012	0.559	Down	TCGGGGATCATCATGTCACGAGA
**chi-miR-301b**	0.42	-1.25	0.018	0.559	Down	CAGTGCAATGATATTGTCAAAGC
**chi-miR-130b-5p**	0.41	-1.30	0.024	0.621	Down	ACTCTTTCCCTGTTGCACTACT
**chi-miR-223-5p**	0.35	-1.50	0.007	0.559	Down	TGTGTATTTGACAAGCTGAGTTG
**chi-miR-412-5p**	0.33	-1.62	0.001	0.276	Down	TGGTCGACCAGTTGGAAAGTAAT
**chi-miR-665**	0.32	-1.65	0.019	0.559	Down	ACCAGTAGGCCGAGGCCCCT
**chi-miR-223-3p**	0.26	-1.92	0.003	0.511	Down	TGTCAGTTTGTCAAATACCCCA
**chi-miR-496-3p**	0.20	-2.29	0.030	0.671	Down	TGAGTATTACATGGCCAATCT
**chi-miR-106a-3p**	0.11	-3.18	0.047	0.769	Down	TACTGCAATGCAAGCACTTCTT

**Table 2:** The differentially expressed lncRNAs

lncRNAs	Fold Change	log_2_FoldChange	p-value	Up/down	Cufflinks-gene ID	Class-code
**TCONS_00003699**	Inf	Inf	0.004706517	Up	XLOC_002888	u
**TCONS_00055666**	Inf	Inf	0.010807149	Up	XLOC_042144	u
**TCONS_00066337**	Inf	Inf	0.033426028	Up	XLOC_050454	u
**TCONS_00080513**	Inf	Inf	0.030710818	Up	XLOC_061087	u
**TCONS_00148053**	Inf	Inf	0.002143822	Up	XLOC_110768	u
**TCONS_00148514**	76.19620987	6.251647332	0.003516268	Up	XLOC_111117	u
**TCONS_00063833**	68.69470734	6.102127044	0.029434776	Up	XLOC_048464	o
**TCONS_00143844**	52.60107296	5.717020323	0.001761151	Up	XLOC_107655	u
**TCONS_00158176**	48.33903097	5.595116647	2.27E-05	Up	XLOC_118081	u
**TCONS_00129994**	32.29835189	5.013388644	0.015843692	Up	XLOC_097220	u
**TCONS_00067523**	31.87609402	4.994402952	0.032826209	Up	XLOC_051367	u
**TCONS_00062140**	17.92388624	4.16381157	0.011608389	Up	XLOC_047101	u
**TCONS_00011031**	11.4250959	3.51413437	0.01663844	Up	XLOC_008649	u
**TCONS_00144689**	11.38306796	3.50881754	0.000908273	Up	XLOC_108139	u
**TCONS_00088402**	7.928516015	2.987050861	0.003135758	Up	XLOC_067279	u
**TCONS_00061579**	7.079741575	2.8236967	0.001287427	Up	XLOC_046679	u
**TCONS_00041067**	6.941202278	2.795185572	0.023619753	Up	XLOC_031274	u
**TCONS_00102108**	6.93225827	2.793325405	0.020870614	Up	XLOC_077600	u
**TCONS_00108242**	6.807163735	2.767053812	0.039646296	Up	XLOC_082113	u
**TCONS_00081414**	6.661186743	2.735779228	0.028152774	Up	XLOC_061778	o
**TCONS_00138804**	5.600968309	2.485676265	0.03168414	Up	XLOC_103830	u
**TCONS_00111220**	5.349384894	2.419373011	0.044515453	Up	XLOC_084271	u
**TCONS_00091259**	5.185906859	2.374596295	0.010227508	Up	XLOC_069374	u
**TCONS_00128617**	4.959484256	2.310190101	0.00530008	Up	XLOC_096084	u
**TCONS_00144434**	4.107474541	2.038251633	0.010156005	Up	XLOC_107993	u
**TCONS_00111216**	4.088620971	2.031614326	0.038218475	Up	XLOC_084271	u
**TCONS_00001644**	3.736372488	1.901638288	0.01127339	Up	XLOC_001306	u
**TCONS_00110456**	3.725766119	1.897537114	0.01108338	Up	XLOC_083717	u
**TCONS_00091228**	0.094906909	-3.39734307	0.049702596	Down	XLOC_069347	i
**TCONS_00146083**	0.073915782	-3.75797377	0.001717991	Down	XLOC_109192	u
**TCONS_00055279**	0.051221771	-4.28709905	0.015184126	Down	XLOC_041859	u
**TCONS_00122260**	0	0	0.004657073	Down	XLOC_091603	o
**TCONS_00132241**	0	0	0.009949405	Down	XLOC_098926	u

**Figure 2:**
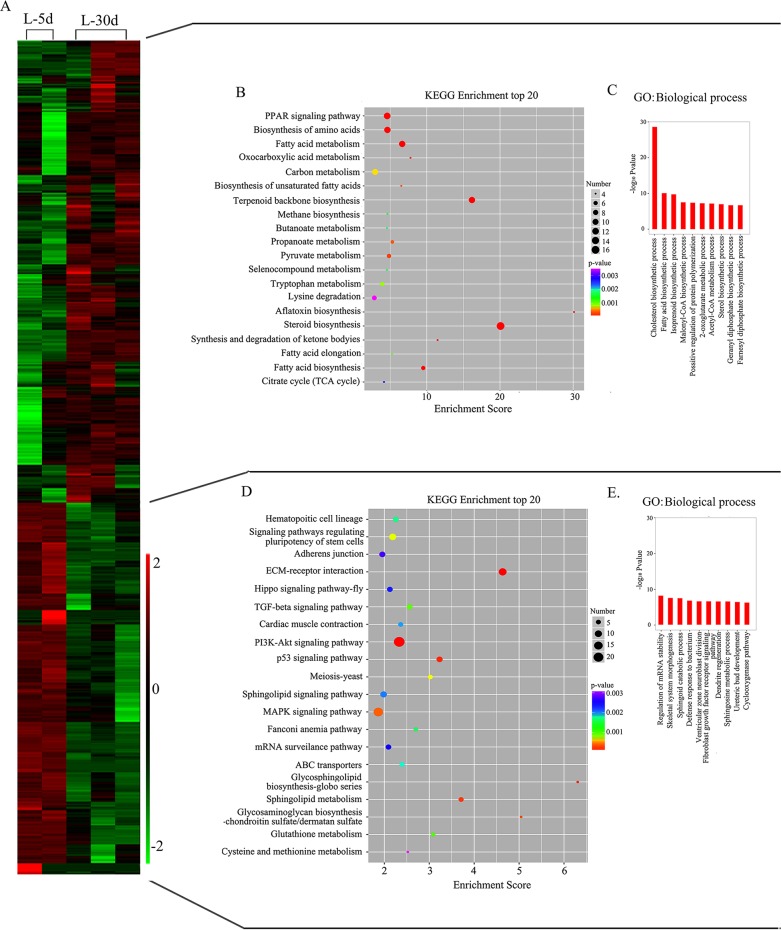
Mammary gland gene expression profile at L-5 d and L-30 d with differentially enriched canonical functions related to lactation process at L-30 d **(A)** Heat map for the gene expression profile. **(B)** KEGG pathway analysis for up-regulated genes at L-30 d. **(C)** Biological process in GO enrichment for up-regulated genes at L-30 d. **(D)** KEGG pathway analysis for down-regulated genes at L-30 d. **(E)** Biological process in GO enrichment for down-regulated genes at L-30 d.

Moreover, we performed GO and KEGG pathway analyses for the differentially expressed mRNAs for down- or up-regulated genes separately (Figure 2; Supplementary Table 7, Supplementary Table 8, Supplementary Table 9 and Supplementary Table 10). In the up-regulated gene cluster, the PPAR signalling pathway, biosynthesis of amino acid, fatty acid biosynthesis, fatty acid metabolism, steroid biosynthesis, and TCA cycle were the most enriched pathways, with a greater number of genes in the top 20 KEGG pathways (Figure 2B; Supplementary Table 7). In the GO biological process category, the top 10 classes were the cholesterol biosynthetic process, fatty acid biosynthetic process, isoprenoid biosynthetic process, malonyl-CoA biosynthetic process, positive regulation of protein polymerization, 2-oxoglutarate metabolic process, acetyl-CoA metabolic process, sterol biosynthetic process, geranyl diphosphate biosynthetic process and farnesyl diphosphate biosynthetic process, which matched well with the KEGG pathways (Figure 2C; Supplementary Table 9). However, the PI3K-AKT signalling pathway, MAPK signalling pathway, EMC receptor interaction and p53 signalling pathway were the most enriched pathways with a greater number of genes in the top 20 KEGG pathways of the down-regulated genes (Figure 2D; Supplementary Table 8). Similarly, the top 10 classes in the GO biological process category of the down-regulated genes include regulation of mRNA stability, skeletal system morphogenesis, sphingoid catabolic process, defence response to bacterium, ventricular zone neuroblast division, fibroblast growth factor receptor signalling pathway involved in orbitofrontal cortex development, dendrite regeneration, sphingosine metabolic process, ureteric bud development and cyclooxygenase pathway (Figure 2D; Supplementary Table 10). Our data partially matched early data from bovine mammary gland lactation stages [32], in both bovine milk [33] and goat milk [34]; this may be because of the different stages or different species of animals investigated in the different studies.

### Competitive interactions among coding-RNAs and lncRNAs during lactation

**Figure 3:**
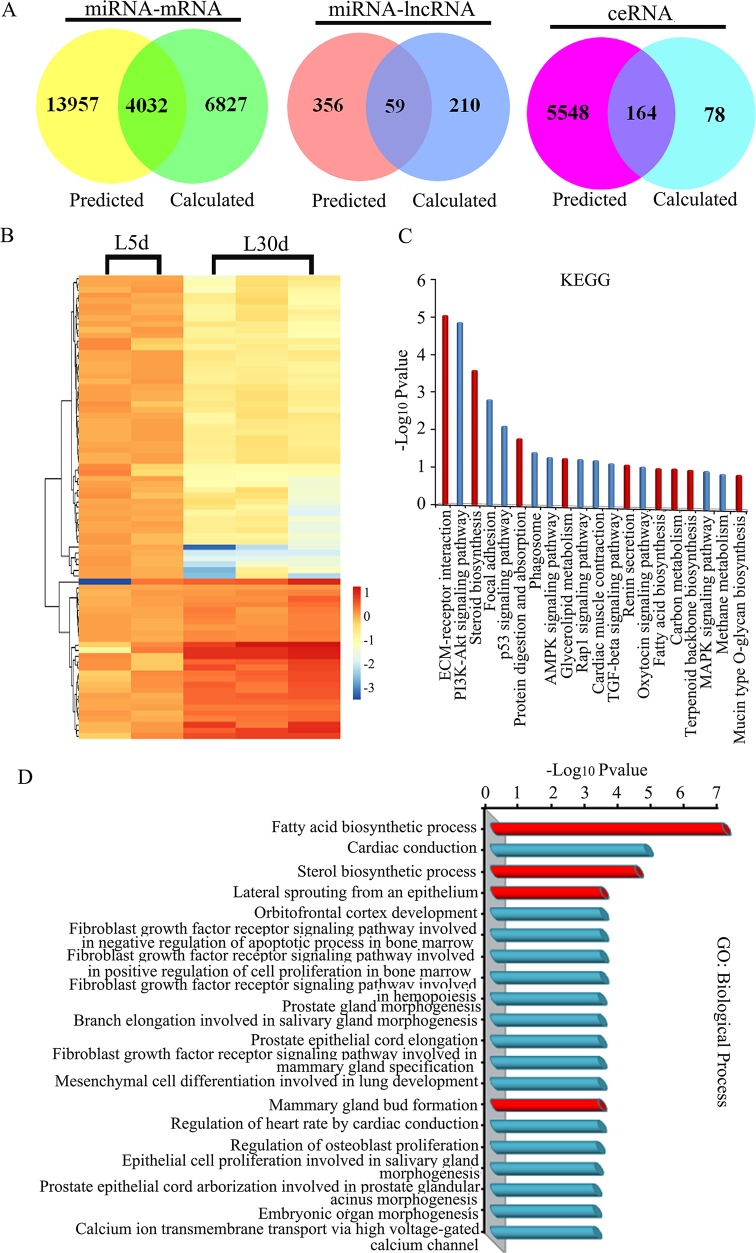
**(A)** Venn diagram showing the overlapping relationships of miRNA-mRNA, miRNA-lncRNA and ceRNA (lncRNA-mRNA). **(B)** Heat map for the differentially expressed ceRNAs. **(C)** KEGG pathway analysis for up-regulated ceRNAs at L-30 d. **(D)** Biological process in GO enrichment for up-regulated ceRNAs at L-30 d.

To reduce background and non-specific interactions, only differentially expressed mRNAs (1150 genes), miRNAs (n =24) and lncRNAs (n = 33) were used to explore target competition during lactation. We used the miRanda algorithm to obtain a prediction of miRNA regulation of protein-coding RNAs (mRNAs) and lncRNAs (see Materials and Methods). It was found that all the differentially expressed genes (1150) were predicted to be regulated by the 24 differentially expressed miRNAs with 17 989 of miRNA recognition elements (MREs; Figure 3A). Then, interactions between miRNAs and mRNAs were predicted based on their correlation in gene expression and 10 859 MREs were found. The shared MREs between predicted MREs based on miRNA binding sites and MREs predicted from the expression were considered as valuable MREs for further ceRNAs searches (see Materials and Methods). The shared number of MREs for miRNAs-mRNAs was 4032 (Figure 3A). Similarly, the shared number of MREs between the predicted MREs (415) and calculated MREs (269) for miRNAs-lncRNAs was 59 (Figure 3A). The shared 4032 miRNAs-mRNAs and 59 miRNAs-lncRNAs were then used to build the ceRNAs. In this investigation, we aimed to investigate lncRNAs as ceRNAs (lncRNA-mRNA pair) in the lactation process. The number of predicted ceRNA based on ceRNA score was 242 from using the shared miRNA-mRNAs and shared miRNA-lncRNAs (Supplementary Table 11). The number of calculated ceRNAs based on the expression of miRNAs, mRNAs and lncRNAs was 5712. The number of shared ceRNA between predicted ceRNAs and calculated ceRNAs was 164, and these were the ceRNAs (lncRNA-mRNA) used in this investigation for further analysis (Figure 3A) [35–37].

### lncRNAs are associated with lactation according to ceRNA interactions

In mammary gland cells, lncRNAs are dynamically expressed and they function during mammary gland development and even in breast cancer [24–28]. However, lncRNAs that act as ceRNAs have not yet been implicated in mammary gland development, especially during the lactation process. In our study, at the transit stage of lactation, 33 lncRNAs were differentially expressed and most of the lncRNAs (n = 28) were up-regulated at L-30 d. As demonstrated by the clustering in Figure 3B, 1/3 of the 164 ceRNAs were up-regulated at L-30 d and 2/3 of the ceRNAs were down-regulated in this phase (Figure 3B). To predict the functions of ceRNAs during lactation, we determined the enrichment of GO terms and KEGG pathway analysis for coding genes directly interacting with lncRNAs. In the GO biological process category, the most significant classes were fatty acid biosynthesis, steroid biosynthesis, lateral sprouting from an epithelium; and in KEGG pathway analysis the most significant pathways included ECM-receptor interaction, steroid biosynthesis, protein digestion and absorption, glycerolipid metabolism, fatty acid biosynthesis and carbon metabolism, which support the findings that lipid metabolism, steroid metabolism and EMC-receptor interaction are important in the lactation process (Figure 3C & 3D) [11–13].

**Figure 4:**
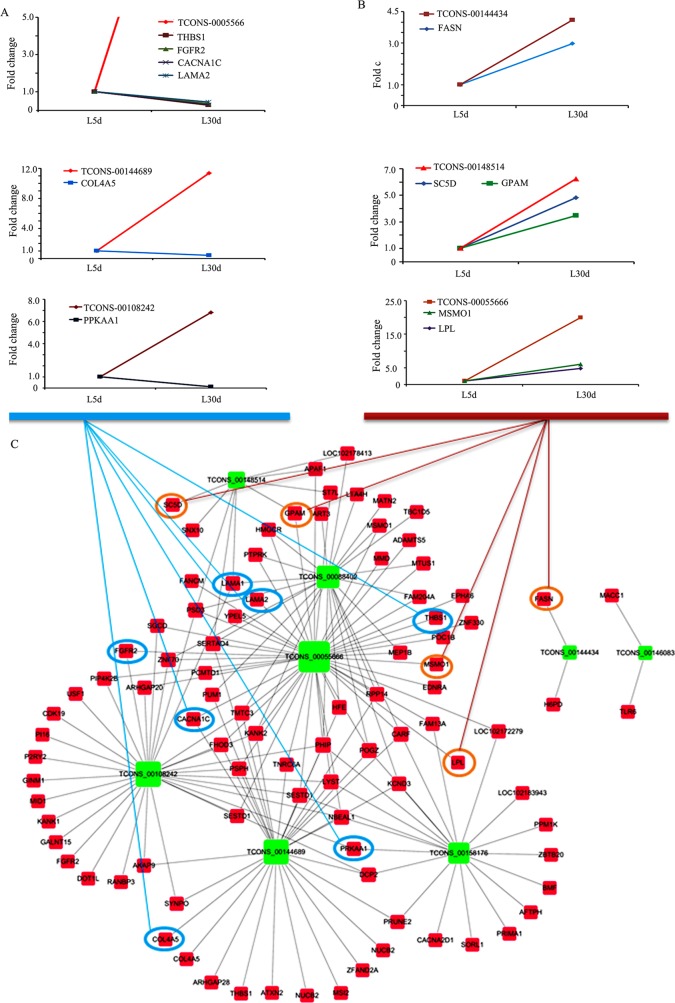
ceRNA (lncRNA-mRNA) interactions and the expression correlation of lncRNA and mRNA **(A)** Expression of three groups of ceRNAs (lncRNA-mRNA) that are negatively correlated during lactation. **(B)** Expression of three groups of ceRNAs (lncRNA-mRNA) that are positively correlated during lactation. **(C)** ceRNA (lncRNA-mRNA) network during lactation.

**Figure 5:**
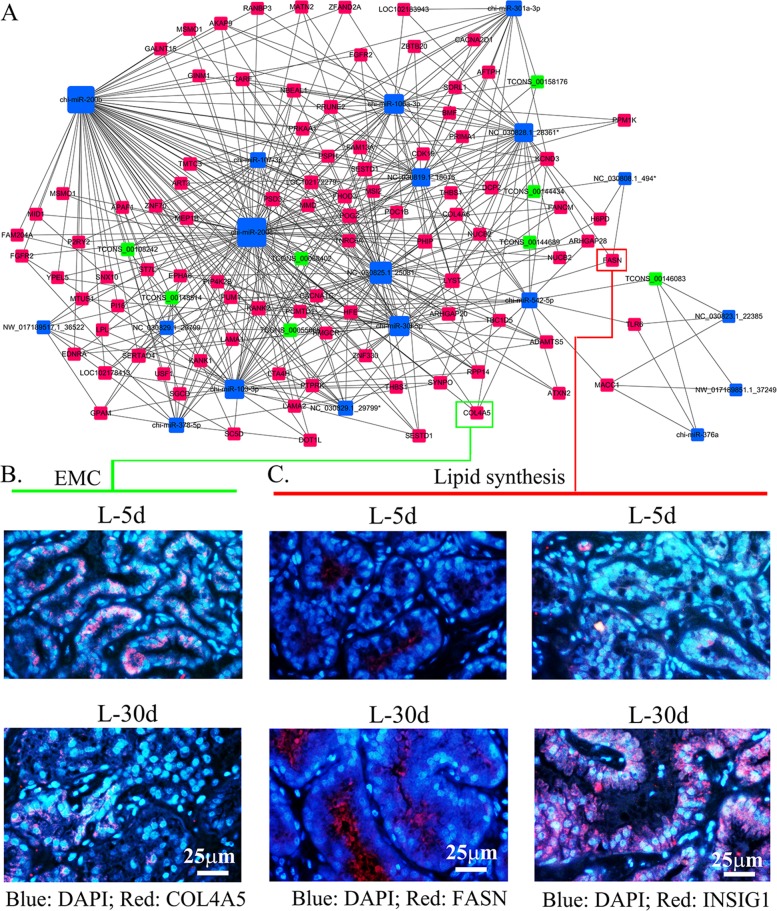
**(A)** Network of ceRNAs (lncRNA-mRNA) and miRNAs. **(B)** The protein level of COL4A5 in mammary gland by IHF analysis. **(C)** The protein levels of FASN and INSIG1 in mammary gland by IHF analysis.

In order to search the interaction of ceRNAs in the ceRNA network, we further analysed ceRNAs by comparing expression levels. We found that lncRNAs TCONS-00055666, TCONS-00144689 and TCONS-00108242 were up-regulated at L-30 d and their ceRNAs paired mRNAs (genes) THBS1, FGFR2, CACNA1C, LAMA2, COL4A5 and PPKAA1 were down-regulated during the same phase. Functions of these genes include EMC-receptor interaction, PI3K-Akt signalling pathway and MAPK signalling pathway (Figure 4A & 4C). This agrees with the mammary gland morphology change stated earlier and also matches the functional changes of these 2 phases [11–13]. On the other hand, lncRNAs TCONS-00144434, TCONS-00148514 and TCONS-00055666 were up-regulated at L-30 d and their ceRNAs paired mRNAs (genes) FASN, LPL, GPAM SC5D and MSMO1 were also up-regulated in this phase. The functions of these genes include fatty acid biosynthesis, sterol biosynthesis and positive regulation of cholesterol storage (Figure 4B & 4C). This agrees with previous findings that the mammary gland produces a continuous supply of milk for the infant and that fatty acids are one of the most important components of milk in this phase [11–13]. The ceRNA network of lncRNA-mRNA was present in Figure 4C, where the nodes represented mRNAs or lncRNAs and the edges represented their competing interactions. LncRNA TCONS_00055666 had the most number of mRNAs as ceRNA, then less number of mRNAs were as the ceRNAs for lncRNAs TCONS_0088402, TCONS_00108242, TCONS_00144689, TCONS_00158176 and TCONS_ 00148514. There were just 2 mRNAs as ceRNAs for lncRNA TCONS_00144434 or TCONS_00146083. The network of ceRNA (lncRNA-mRNA) and miRNA was shown in Figure 5A, where the nodes represented mRNAs, lncRNAs or miRNAs and the edges represented their competing interactions. miR-200b and miR-200c formed more interaction with mRNA or lncRNA than other miRNAs. These networks suggested that miRNAs, lncRNAs and miRNAs interact together in the lactating mammary gland. They might be involved in the lactation process.

Since, ceRNAs play important roles in lactation, next we further analysed protein levels of a few important regulators in lactation: FASN, INSIG1 and COL4A5, which were found to be differentially expressed. In agreement with ceRNA (lncRNA-mRNA) expression patterns, COL4A5 was down-regulated at L-30 d (Figure 5A & 5B), while FASN and INSIG1 were up-regulated at L-30 d (Figure 5A & 5C) [13].

## DISCUSSION

In this investigation, we determined the ceRNAs of lncRNA-mRNA during different phases of lactation through the MREs in miRNAs. Over the past decades, miRNAs have been extensively explored in many organisms, tissues and even in mammary gland development. Studies suggest that miRNAs play important roles in different stages of mammary gland development [38, 39]. It was found that some important miRNAs were up-regulated during lactation [38–40]. In our current investigation, we found 9 miRNAs were up-regulated at L-30 d compared with early lactation (L-5 d).

Compared to miRNAs, only a few studies have explored lncRNAs in mammary gland development. First reported was lncRNA *Pinc*, which is a pregnancy-induced lncRNA [41]. Other lncRNAs related to mammary gland development include *Zfas* [42], *HOTAIR and GAS5-AS1* [40], *Ror* and *Neat1* [28]. Taken together, these studies suggest that lncRNAs as well as miRNAs play a crucial role in regulating mammary epithelial cells. It has been reported that the expression of lncRNAs is more tissue-specific than protein coding genes [43], and the competitive interactions of lncRNAs are more tissue-specific [44]. These were the fundamental principles used in our studies and we identified 33 lncRNAs that were differentially expressed in the 2 phases of lactation and 28 of them were up-regulated at L-30 d.

MicroRNAs are commonly considered as active regulatory elements to decrease the stability of target RNAs or to block their translation [35]. However, Poliseno *et al*., demonstrated that pseudogenes acting as microRNA competitors thereby actively competed with their ancestral protein-coding genes for the same pool of microRNAs through sets of conserved MREs [45]. This suggests that non-coding RNA targets can cross-talk through their ability to compete for microRNA binding [35, 45]. Therefore, MREs can be used by the transcripts to actively communicate with each other and to regulate their respective expression levels [35]; furthermore, MREs may functionalize the entire mRNA dimension through cross-talking ceRNAs and even ceRNA networks [35].

In previous studies, only lncRNA and mRNA were analysed in the same samples, and miRNAs were computed from other databases to determine the ceRNA of lncRNA-mRNA, mRNA-mRNA and lncRNA-lncRNA [44]. In the current study, we sequenced mRNA, miRNA and lncRNA in the same samples at 2 phases of lactation. All data were co-expressed together from the same samples. Furthermore, we constructed the ceRNA and ceRNA network using the expression data for the same samples. This is the first study to be performed in this way. Even though a lower number of ceRNAs were found in our study, the data is more accurate and more reliable because all the expression of mRNA, miRNA and lncRNA were correlated. In total, 164 ceRNAs were constructed in this study and further functional analysis suggested that the dynamic expression and regulation pattern were related to lactation. As early studies indicate that highly expressed genes in particular developmental stages or cell types are functionally active, our data showed that 1/3 of the ceRNAs were up regulated during the mature lactation phase with the functions of lipid, protein, carbon, amino acid synthesis and metabolism. This correlates with the function of the mature lactation phase, i.e. the continuous production of a large amount of milk rich in protein, lipids, amino acids and other nutrients. Furthermore, 2/3 of ceRNAs were up-regulated in early lactation with functions in PI3K-AKT pathways or ECM-receptor interactions. This correlated with the function of this lactation phase, i.e. to prepare the mammary gland for full lactation. Therefore, the results indicated that ceRNAs work synergistically during different developmental stages to regulate specific functions, particularly to control lactation in the mammary gland.

In summary, by applying ceRNA network analysis of transcriptomes (mRNA, miRNA, lncRNA) obtained during lactation, this study provides a novel approach for understanding gene functionality in this biological process. The investigation yielded many new insights into the structure of molecular pathways in the lactation process. Analysing ceRNA interactions in the context of lactation may provide insights into the regulation of lactation. Moreover, this study suggests that ceRNAs (lncRNA-mRNA) may be involved in lactation process.

## MATERIALS AND METHODS

### Mammary gland tissue collection and morphological analysis

This investigation was performed in strict accordance with the recommendations in the Guide for the Care and Use of Laboratory Animals of the National Institutes of Health. The protocol was approved by the Committee on the Ethics of Animal Experiments of Qingdao Agricultural University IACUC (Institutional Animal Care and Use Committee). Six 2-year-old Laoshan dairy goats, one of 4 native Chinese dairy goat breeds, were used in this study. Goat mammary gland tissue from lactation day 5 (L-5 d; early lactation) and lactation day 30 (L-30 d; mature lactation) were surgically collected and frozen immediately in liquid nitrogen for further analysis (3 animals/group) [46]. Part of the mammary gland samples were fixed in 10% neutral formalin and then paraffin embedded. Subsequently, 5-μm sections were prepared and stained with hematoxylin and eosin (H&E) for the analysis of morphological changes [47, 48].

### Sequencing

Total RNA was extracted from individual mammary gland samples and cDNA libraries were constructed. For small RNA cDNA library, total RNA (about 1 μg) was ligated with *RNA 3’ Adapter and 5’ Adapter*, then the ligated RNAs were reversely transcribed to cDNAs using RT primers. The cDNAs were amplified by PCR, purified by gel electrophoresis. The quality was determined by Agilent 2100 chip. For RNA-seq analysis library (mRNA and lncRNA), total RNA was purified to remove rRNA by Ribo-Zero™ rRNA Removal Kit, then RNA was fragmented. The fragmented RNA was reversely transcribed into the first strand cDNA by TruSeq® Stranded kit, then DNA polymerase I and RNAase H were used to form the double strands cDNA. The double stranded cDNA was adenylate 3’ Ends and ligated Adapters. Then the RNA was amplified by PCR and purified to form the cDNA library. Libraries were sequenced using the Illumina HiSeq 2500 platform with the 90-bp pair-end sequencing strategy for total RNA sequencing and Illumina-HiSeqXTen platform for small RNA sequencing. The original image data generated by the sequencing machine were converted into sequence data by Illumina pipeline CASAVA v1.8.2 and then subjected to standard QC criteria to remove the contaminant following parameters [44]:

1. Reads aligned to adaptors or primers with no more than 2 mismatches.
2. Reads with *>*10% unknown bases (*N* bases).
3. Reads with *>*50% low quality bases (quality value ≤ 5) in one read.

### Read mapping and gene expression analysis

The reference genome, transcriptome and annotation reference for the goat is located on the NCBI platform (https://www.ncbi.nlm.nih.gov/genome/?term=Capra%20aegagrus%20hircus/genome.fa.gz; https://www.ncbi.nlm.nih.gov/genome/?term=Capra%20aegagrus%20hircus/transcriptome.fa.gz; https://www.ncbi.nlm.nih.gov/genome/?term=Capra%20aegagrus%20hircus/gff.gz). Clean paired-end reads were aligned to the reference genome using TopHat (version 2.0.6) [49]. The transcriptome of each sample was constructed using Cufflinks (version 2.0). Transcripts *>*200 nt were identified as lncRNAs if they did not overlap with known genomic annotations from the database [49]. miRNAs were analysed using Bowtie software and the miRBase database. Next, the reads per kilo base of model per million base pairs sequenced (RPKM) was used to quantify the expression levels of a gene or lncRNA [44] and transcript per million (TPM) was used to determine the expression levels of miRNA [37]. The difference between different group was determined by DEGseq software (Fold Change≥2.00 and *FDR*≤ 0.001).

### miRNA target prediction

miRanda database was used with the default parameters to identify conserved miRNA target sites in the 3’UTR of coding transcripts and full-length lncRNA transcripts [37]. If multiple annotated 3’UTR*/*lncRNA sequences were found for a coding*/*lncRNA gene, the longest was used in the analysis. These were the predicted pairs for miRNA target of the differentially expressed miRNAs, mRNAs and lncRNAs in the 2 goat mammary gland phases studied.

### Construction of the ceRNA NET work related to lactation phase [44, 50]

#### Overview of the processes used to identify ceRNA interaction pairs

Based on the expression levels of mRNAs, miRNAs and lncRNAs, Pearson's correlation coefficient and P-value were calculated for miRNA-target. Negatively correlated pairs with a P-value <0.05 and Pearson's correlation coefficient >0.7 were selected for further analysis. These were the predicted pairs for miRNA-mRNA, miRNA-lncRNA and mRNA-lncRNA of the differentially expressed miRNAs, mRNAs and lncRNAs in the 2 goat mammary gland phases. Subsequently, shared pairs from the predicted pairs from binding sites and the predicted pairs from the expression of mRNA, lncRNA and miRNA were used for further analysis.

Based on the principle for ceRNA prediction, shared pairs of miRNA-mRNA and miRNA-lncRNA were used to predict ceRNA score according to the following formula [50].

$$\text{ceRNA}\_\text{score}=\frac{\#\text{MRE}\_\text{for}\_\text{share}\_\text{miRNA}}{\#\text{MRE}\_\text{for}\_\text{lncRNA}\_\text{miRNA}}$$

P-value was calculated as follows [50]:

$$p=\overset{\min\;(m_{p},m_{n})}{\underset{i=m_{c}}{\sum }}\frac{\left(\begin{array}{c} m_{n}\\ i \end{array}\right)\left(\begin{array}{c} N_{T}-m_{n}\\ m_{p}-i \end{array}\right)}{\left(\begin{array}{c} M_{T}\\ m_{p} \end{array}\right)}$$

Where M_T_: total number of miRNAs; m_p_: number of miRNA correlated with the mRNA; m_n_: number of miRNA correlated with the lncRNA; m_c_: number of shared miRNA.

The shared pairs from the predicted pairs of lncRNA-mRNA based on the expression of lncRNA and mRNA (Pearson's correlation coefficient) and the predicted pairs of lncRNA-mRNA based on ceRNA score principle were then considered as the true ceRNAs (lncRNA-mRNA).

#### Enriched functional categories for ceRNA [44]

The functional annotations of genes for ceRNAs (lncRNA-mRNA) were obtained from the GO and KEGG databases

#### Construction of the ceRNA network

The ceRNA network was constructed by assembling all the ceRNA pairs, where the nodes represented mRNAs or lncRNAs and the edges represented their competing interactions. The network for ceRNA and miRNAs was also formed, where the nodes represented miRNAs and the edges represented the ceRNA and miRNA interactions

### Real-time quantitative RT-PCR

The procedure for mRNA q-RT-PCR was reported in our early publications [51]. MiRNA q-RT-PCR was performed using the Hairpin-it™ miRNA RT-PCR (probe) Quantitation kit from GenePharma Co., Ltd (Shanghai, China) following the manufacturer's instructions, as described in our recent publication [52]. Similarly, lncRNA q-RT-PCR was performed using the Hairpin-it™ lncRNA RT-PCR (probe) Quantitation kit from GenePharma Co., Ltd following the manufacturer's instructions. RNA from mammary gland tissues was extracted using TRIzol Reagent (Invitrogen Corp., Carlsbad, CA, USA) and purified using an RT2 qPCR-Grade RNA Isolation Kit from SABiosciences Co., Ltd (MD, USA). Total RNA was quantified using a Nanodrop 3300 (ThermoScientific, DE, USA). The quality of RNA was controlled by the A260:A280 ratio being greater than 2.0 and confirmed by electrophoreses, with a fraction of each total RNA sample with sharp 18S and 28S ribosomal RNA (rRNA) bands as reported in our recent publication [52]. One microgram of total RNA was used to make the first strand cDNA in 20 μl. The program for the reaction of miRNA and lncRNA was 25°C for 30 min, 42°C for 30 min, 85°C for 5 min, then 4°C or on ice. The qPCR was performed with the Roche LightCycler 480 (Roche, Germany) and the reaction was as follows: Step 1, 95°C for 3 min; Step 2, 40 cycles of 95°C for 12 s; 62°C for 40 s. The primer sets for mRNA, miRNA and lncRNA are given in Supplementary Table 12, 13 and 14. Three independent experimental samples were analysed [52]. q-RT-PCR was statistically analysed using proprietary software from SABiosciences online support (www.SABiosciences.com).

### Immunofluorescence staining

Mammary gland sections (5 μm) were prepared and subjected to antigen retrieval and immunostaining as previously described [46]. Briefly, sections were first blocked with normal goat serum in PBS, followed by incubation (1:150 in PBS-1% BSA) with rabbit anti-FASN Ab (Cat no.: bs-1498R) and rabbit anti-INSIG1 Ab (Cat no.: bs-5074R) from Beijing Biosynthesis Biotechnology Co., Ltd (Beijing, China) and rabbit anti-COL4A5 Ab (Cat no.: sc-9302) from Santa Cruz Co., Ltd (CA, USA) overnight at 4°C. The following morning, after 3 washes with PBS Tween 20 (0.5%), the slides were incubated with Alexa Fluor 546 goat anti-rabbit IgG (1:200) for 30 min in darkness at RT. The negative control samples were incubated with secondary antibody and without primary antibody. Slides were washed thrice with PBS Tween-20 and then incubated with DAPI (4.6-diamidino-2-phenylindole hydrochloride, 100 ng/ml) as a nuclear stain for 5 min. After a brief wash with ddH_2_O, the slides were covered with an anti-fading mounting medium from Vector Co., Ltd (CA, USA). Fluorescent images were obtained using a Leica Laser Scanning Confocal Microscope (LEICA TCS SP5 II, Germany) [46].

### Data access

The raw sequencing data generated in this study has been uploaded to the NCBI SRA database with the accession number: PRJNA361394 (http://www.ncbi.nlm.nih.gov/bioproject/PRJNA361394/).

## SUPPLEMENTARY MATERIALS TABLES
























