# Supplementary material for: LncRNA as ceRNAs may be involved in lactation process
Source: Oncotarget. 2017 Aug 24;8(58):98014–28. doi: 10.18632/oncotarget.20439 (PMC5716710; doi:10.18632/oncotarget.20439)
Supplement: Supplementary file 1 [file oncotarget-08-98014-s001.pdf]

LncRNA as ceRNAs may be involved in lactation process

SUPPLEMENTARY MATERIALS

Supplementary Table 1: Data summary of RNA-seq

Sample	Raw reads	Raw bases	Clean reads	Clean bases	Valid ratio (base)	Q30 (%)	GC content (%)
Sample_L30d_1	155505456	19438182000	153035162	19123263832	98.37%	95.77%	48.50%
Sample_L30d_2	160032308	20004038500	157508272	19682245827	98.39%	95.82%	48.00%
Sample_L30d_3	159660662	19957582750	157308658	19657406671	98.49%	95.88%	49.50%
Sample_L5d_1	169272922	21159115250	166369552	20789336783	98.25%	95.72%	47.50%
Sample_L5d_2	149416418	18677052250	146610704	18319956572	98.08%	95.51%	47.00%
Sample_L5d_3	141552176	17694022000	139143930	17387375769	98.26%	95.79%	48.00%

Note: The data is read1+read2.

Supplementary Table 2: Data summary for the alignment to the genome (RNA-seq)

See Supplementary File 1

Supplementary Table 3: Summary clean reads for small RNA sequencing

Sample	raw_reads	reads_trimmed_length	reads_trimmed_Q20	reads_trimmed_N	clean_reads	clean_reads_uniq
Sample_L30d_1	14518192	713518	3140	3348	13798186	409936
Sample_L30d_2	16167627	749171	3006	3843	15411607	379056
Sample_L30d_3	14484629	815427	2611	3330	13663261	452993
Sample_L5d_1	13920183	592590	2812	3350	13321431	250502
Sample_L5d_2	14579032	466015	2852	3648	14106517	292042
Sample_L5d_3	15957232	596424	3471	3842	15353495	402009

Supplementary Table 4: Summary for the alignment to the genome (small RNA sequencing)

Sample	Reads	Aligned_reads	Aligned(%)
Sample_L30d_1	13798186	13332465	96.62%
Sample_L30d_1_uniq	409936	335259	81.78%
Sample_L30d_2	15411607	14675477	95.22%
Sample_L30d_2_uniq	379056	235967	62.25%
Sample_L30d_3	13663261	13263218	97.07%
Sample_L30d_3_uniq	452993	378604	83.57%
Sample_L5d_1	13321431	12788374	95.99%
Sample_L5d_1_uniq	250502	176404	70.42%
Sample_L5d_2	14106517	13516648	95.81%
Sample_L5d_2_uniq	292042	198281	67.89%
Sample_L5d_3	15353495	14899850	97.04%
Sample_L5d_3_uniq	402009	339971	84.56%

Supplementary Table 5: Novel miRNAs

See Supplementary File 2

Supplementary Table 6: The differentially expressed novel miRNAs

See Supplementary File 3

Supplementary Table 7: The KEGG pathway analysis for up-regulated genes

See Supplementary File 4

Supplementary Table 8: The KEGG pathway analysis for down-regulated genes

See Supplementary File 5

Supplementary Table 9: The GO enrichment for up-regulated genes

See Supplementary File 6

Supplementary Table 10: The GO enrichment for down-regulated genes

See Supplementary File 7

Supplementary Table 11: Predicted ceRNAs

See Supplementary File 8

Supplementary Table 12: mRNA primer sets

See Supplementary File 9

Supplementary Table 13: miRNA primer sets

See Supplementary File 10

Supplementary Table 14: lncRNA primer sets

See Supplementary File 11
