# Supplementary material for: LncRNA as ceRNAs may be involved in lactation process
Source: Oncotarget. 2017 Aug 24;8(58):98014–28. doi: 10.18632/oncotarget.20439 (PMC5716710; doi:10.18632/oncotarget.20439)
Supplement: Supplementary file 2 [file oncotarget-08-98014-s002.doc]

Supplementary Table 2: Data summary for the alignment to the genome (RNA-seq)

| **样本名**  **比对信息** | Sample_L30d_1 | Sample_L30d_2 | Sample_L30d_3 | Sample_L5d_1 | Sample_L5d_2 | Sample_L5d_3 |
| --- | --- | --- | --- | --- | --- | --- |
| Total reads | 153035162 | 157508272 | 157308658 | 166369552 | 146610704 | 139143930 |
| Total mapped | 136396325  (89.13%) | 143324459  (90.99%) | 143318678  (91.11%) | 153974960  (92.55%) | 135337335  (92.31%) | 127351896  (91.53%) |
| Multiple mapped | 22022592  (14.39%) | 20705271  (13.15%) | 23267861  (14.79%) | 15082867  (9.07%) | 11340326  (7.73%) | 15005734  (10.78%) |
| Uniquely mapped | 114373733  (74.74%) | 122619188  (77.85%) | 120050817  (76.32%) | 138892093  (83.48%) | 123997009  (84.58%) | 112346162  (80.74%) |
| Read-1 | 58121601  (37.98%) | 62126028  (39.44%) | 60644939  (38.55%) | 70075584  (42.12%) | 62560460  (42.67%) | 56745489  (40.78%) |
| Read-2 | 56252132  (36.76%) | 60493160  (38.41%) | 59405878  (37.76%) | 68816509  (41.36%) | 61436549  (41.90%) | 55600673  (39.96%) |
| Reads map to '+' | 56649156  (37.02%) | 60906062  (38.67%) | 59778962  (38.00%) | 69371503  (41.70%) | 62013710  (42.30%) | 56026030  (40.26%) |
| Reads map to '-' | 57724577  (37.72%) | 61713126  (39.18%) | 60271855  (38.31%) | 69520590  (41.79%) | 61983299  (42.28%) | 56320132  (40.48%) |
| Non-splice reads | 79579585  (52.00%) | 86314193  (54.80%) | 88018012  (55.95%) | 110762290  (66.58%) | 105053038  (71.65%) | 87438468  (62.84%) |
| Splice reads | 34794148  (22.74%) | 36304995  (23.05%) | 32032805  (20.36%) | 28129803  (16.91%) | 18943971  (12.92%) | 24907694  (17.90%) |
| Reads mapped in proper pairs | 97718470  (63.85%) | 104748478  (66.50%) | 107897440  (68.59%) | 117603722  (70.69%) | 102657254  (70.02%) | 95816064  (68.86%) |
